# Modulation of Intravenous Immunoglobulin Aggregation, Subvisible Particle Formation, and Viscosity by Acetylated Amino Acids

**DOI:** 10.3390/pharmaceutics18050544

**Published:** 2026-04-28

**Authors:** Arun Mainali, Binod Lamichhane, Hyo Ri Lee, Ki Hyun Kim, Seong Hoon Jeong, Nam Ah Kim

**Affiliations:** 1 College of Pharmacy, Mokpo National University, Muan 58554, Republic of Korea; arunparty123@mokpo.ac.kr (A.M.); way2binod@mokpo.ac.kr (B.L.); kihyunkim@mnu.ac.kr (K.H.K.); 2 Department of Biomedicine, Health & Life Convergence Sciences, BK21 Four, Biomedical and Healthcare Research Institute, Mokpo National University, Muan 58554, Republic of Korea; 3 College of Pharmacy, Yonsei University, Incheon 21983, Republic of Korea; hyori@yonsei.ac.kr

**Keywords:** N-acetyl arginine, N-acetyl histidine, protein aggregation, viscosity, protein–protein interactions, protein formulation

## Abstract

**Background:** Arginine and related amino acids are widely used to suppress protein aggregation, thereby affecting stability, manufacturability, and therapeutic performance. However, their effectiveness remains limited, necessitating the exploration of alternative strategies. Previous studies have shown that N-acetyl-L-arginine (NA-Arg) can improve protein stability; however, the potential of other N-acetylated amino acids has not been fully explored. **Methods:** This study aimed to investigate the effects of multiple N-acetylated amino acids as alternative excipients on aggregation, colloidal stability, and viscosity in intravenous immunoglobulin (IVIG) formulations. Dynamic light scattering (DLS) was used to evaluate diffusion behavior and aggregation tendencies, while complementary analyses were performed using size-exclusion chromatography (SEC) and flow-imaging microscopy (FI). **Results:** Overall, N-acetylation of amino acids improved colloidal stability, shifting the kD values from −5.87 to 6.83 mL/g for arginine and from −8.17 to 16.22 mL/g for histidine, and increased the aggregation onset temperature (Tagg) to above 60 °C. Among the tested compounds, N-acetyl-L-histidine (NA-His) showed the most favorable results, increasing the monomer proportion by approximately 4%, reducing high-molecular-weight species to below 2%, and producing a greater than 10-fold decrease in subvisible particles relative to histidine hydrochloride after 5 days of agitation. At 50 mM, both NA-His and NA-Arg reduced the viscosity of highly concentrated 200 mg/mL IVIG formulations, with NA-His exhibiting the lowest viscosity (7.24 ± 0.12 mPa·s). Protein–protein interaction and surface charge analyses indicated improved colloidal stability relative to parent amino acids, attributable to the presence of the acetyl group. **Conclusions:** These findings support the potential of N-acetylation as a strategy to modulate interaction-driven instability and suggest NA-His as a promising candidate excipient for stabilizing highly concentrated therapeutic proteins at acidic pH.

## 1. Introduction

Protein-based biopharmaceuticals are pivotal in the modern management of numerous diseases, including cancer, autoimmune disorders, metabolic diseases, and rare genetic conditions [1,2]. Their exceptional therapeutic efficacy and high target specificity have led to rapid market growth, positioning them as the highest-grossing therapeutics, with sales reaching $387.03 billion by 2023 [3]. A key factor in their market success is the advancement of subcutaneous (SC) injection formulations, which offer greater convenience and improved patient adherence compared to intravenous administration. Nonetheless, the limitation on injection volume for SC delivery necessitates the use of high-concentration antibody formulations. This clinical necessity has presented substantial challenges in managing solution viscosity and achieving stable, concentrated protein formulations.

Maintaining the stability of biopharmaceuticals during manufacturing, storage, and delivery is paramount, as any structural changes, including misfolding, unfolding, unwanted modifications, or aggregation, can significantly diminish efficacy or introduce adverse effects [4]. Beyond stability, issues related to purification and immunogenicity remain critical. However, for SC administration, the viscosity of formulations at concentrations above 100 mg/mL has become a predominant obstacle, complicating manufacturing processes, injectability, and injection [5,6]. Elevated viscosity not only hinders precise filling and handling during production but also increases the force required for injection, potentially surpassing the technical specifications of standard prefilled syringes or autoinjectors. This may affect patient comfort and constrain feasible dosing. Therefore, approaches to reduce viscosity while preserving protein stability are a key challenge in advancing high-concentration antibody therapeutics for SC administration.

Arginine, a naturally occurring amino acid, is used in protein formulations in its salt forms, most commonly as arginine hydrochloride (Arg·HCl), rather than as a free base. It is recognized as one of the most frequently utilized stabilizers and viscosity-modulating excipients in protein-based biopharmaceutical formulations. Among amino acid excipients reported in FDA-approved injectable products, arginine and its salts have been incorporated into approximately 20 formulations and have been extensively investigated due to their ability to suppress aggregation and reduce viscosity [7,8]. However, its impact varies considerably depending on formulation context and the nature of the counterion. The viscosity-lowering capacity of arginine salts, including arginine hydrochloride and arginine glutamate, is strongly dose-dependent. As protein concentration increases, larger quantities of arginine are often required to achieve suitable viscosity reduction, and arginine hydrochloride has been reported to be less effective than arginine glutamate under certain formulation conditions [9,10]. At these elevated concentrations, osmolality values may exceed the threshold for SC administration tolerability, thus limiting its clinical applicability [7]. These challenges highlight the importance of developing alternative excipients or chemical modifications that provide effective stabilization and viscosity reduction while preserving bioactivity.

Conversely, arginine offers distinct advantages over conventional surfactants such as polysorbates (PS20 and PS80), which mitigate interfacial stress through micellization and adsorption but remain limited in their ability to disrupt monomer-level protein self-association [11,12]. Furthermore, the susceptibility of polysorbates to degradation further raises concerns regarding long-term stability and product quality [13]. Recent studies have demonstrated that protein–polysaccharide-based systems can enhance protein stability and delivery through intermolecular interactions and protective matrix formation [14]. However, these approaches often involve increased formulation complexity and potential challenges in reproducibility and large-scale manufacturing. Consequently, small-molecule excipients remain attractive due to their simplicity, robustness, and compatibility with established pharmaceutical processes.

The other amino acids have been studied for their potential to suppress protein aggregation. Histidine, for example, functions both as a widely used buffering agent and has been authorized by the FDA for application in high-concentration protein formulations [15]. However, at elevated concentrations, histidine may contribute to solution discoloration and promote aggregation when stored at higher temperatures (40 °C), particularly in stainless steel containers or after repeated freeze–thaw cycles [16]. Amino acid salts, particularly in the hydrochloride form, such as histidine, lysine, and arginine, have similarly been shown to decrease the viscosity of concentrated IgG1 solutions by governing protein–protein interactions [10,17]. Lysine, particularly in the hydrochloride form, has similarly been shown to decrease the viscosity of simplified model protein such as concentrated albumin solutions (50 mM to 200 mM) by interrupting protein–protein interactions [18]. However, albumin represents a single, well-defined small protein, and while it shares fundamental physicochemical properties common to proteins, it does not capture the compositional heterogeneity and collective interaction behavior characteristic of antibodies.

In addition to these native amino acids, various chemical modifications have garnered increased interest. Specifically, either removal or N-acetylation of the α-amino group in arginine has been reported to enhance colloidal stability relative to arginine monohydrochloride, particularly in acidic environments, by reducing disruptions to protein conformational stability [19]. By converting the positively charged amino group into a neutral amide, N-terminal acetylation changes electrostatic interactions, interferes with salt-bridge formation, and consequently influences protein folding and stability [20]. Importantly, N-acetyl-L-arginine (NA-arginine) has demonstrated an ability not only to suppress stress-induced particle generation but also to reduce thermal destabilization in formulations such as intravenous immunoglobulin (IVIG) and etanercept [21].

Collectively, these findings highlight the potential of N-acetylation as a versatile strategy for protein stabilization and viscosity reduction, without compromising formulation compatibility [22,23]. Previous studies on amino acid excipients have primarily focused on monoclonal antibody systems, providing general insight into protein solutions; however, heterogeneous protein systems remain less explored. N-acetyl-L-arginine has previously been demonstrated to exhibit stabilizing effects compared with arginine hydrochloride; however, the role of other acetylated amino acids remains largely unexplored. On this basis, the present study was designed to expand the understanding of amino acid-based stabilization by evaluating the effects of multiple acetylated amino acids. In particular, this study examines whether N-acetylation of the α-amino group enhances protein stability beyond conventional amino acids, thereby addressing this knowledge gap. The study focuses on the comparative effects of non-acetylated amino acids and their acetylated counterparts on protein stability under agitation and thermal stress conditions. IVIG was chosen due to its high concentration and compositional heterogeneity, which provide a sensitive model for evaluating intermolecular interactions and formulation stability [24]. While monoclonal antibodies are more uniform, the underlying mechanisms governing protein–protein interactions are shared. To quantify monomer preservation and subvisible particulate content after agitation-induced stress, size-exclusion chromatography (SEC) and flow-imaging microscopy (FI) were utilized, while colloidal stability was analyzed via dynamic light scattering (DLS). Additionally, solution rheology at elevated protein concentrations was assessed to determine its viscosity.

## 2. Materials and Methods

### 2.1. Materials

A model drug, IVIG (IVglobulin SN Inj, 10% solution, ~100 mg/mL IgG) stabilized with 18.8 mg/mL glycine, was sourced from GC Biopharma Corp. (Yongin-si, Gyeonggi, Republic of Korea). L-arginine monohydrochloride was obtained from Pfanstiehl Inc. (Waukegan, IL, USA); N-acetyl-L-histidine monohydrate was acquired from Tokyo Chemical Industry Co., Ltd. (Tokyo, Japan); and glycine hydrochloride was purchased from Daejung Chemicals (Siheung-si, Gyeonggi-do, Republic of Korea). L-lysine monohydrochloride, L-cysteine hydrochloride, L-histidine hydrochloride monohydrate, N-acetyl-L-arginine, N-acetyl-L-lysine, N-acetyl-L-cysteine, and N-acetyl-glycine were obtained from Sigma-Aldrich (St. Louis, MO, USA). Phosphate-buffered saline (10× PBS) was obtained from Dyne-Bio (Seongnam-si, Gyeonggi-do, Republic of Korea). Acetic acid and sodium acetate trihydrate were obtained from Sigma-Aldrich. All other reagents used were of analytical grade.

### 2.2. Sample Preparation

To prepare test samples, 100 mg/mL IVIG was diluted to obtain a series of 10 mg/mL IVIG solutions, each formulated with varying concentrations of amino acids and their acetylated derivatives. A 25 mM sodium acetate buffer (pH 4) was used as the common formulation medium for all samples to ensure a consistent ionic strength across all experimental conditions. Stock solutions of each amino acid and its acetylated form (167 mM) were prepared using the same sodium acetate (pH 4) buffer. The pH of all solutions was adjusted using dilute hydrochloric acid (0.1 N HCl) or sodium hydroxide (0.1 M NaOH), and verified using a calibrated pH meter. The stock solutions were subsequently diluted with the same buffer to obtain intermediate working concentrations (55.55 mM). Briefly, each buffer containing the specified amino acid additive at the target concentration was mixed with 100 mg/mL IVIG at a 1:9 (*v*/*v*) ratio, resulting in final samples containing 10 mg/mL IVIG and additives at concentrations of 50 mM or 150 mM, depending on the experimental condition.

To achieve an IVIG concentration of up to 200 mg/mL for viscosity measurement, the IVIG solution was freeze-dried. 50 mg/mL IVIG containing 1% (*w*/*v*) sucrose as a cryoprotectant was prepared from 100 mg/mL IVIG by mixing in a 1:1 ratio with 25 mM sodium acetate buffer containing 2% (*w*/*v*) sucrose (pH 4). 4 mL of the prepared sample was dispensed into sterilized 10 mL glass vials before freeze-drying. The lyophilization process was performed using a pilot-scale shelf freeze dryer (Lyoph-Pride 10, IlshinBiobase, Dongducheon-si, Republic of Korea). Pre-freezing occurred at −40 °C for 3 h, followed by holding at −40 °C for an additional 3 h under 700 mtorr. Primary drying was performed in two steps: (i) at −40 °C and 40 mtorr for 15 h, followed by (ii) at −10 °C and 500 mtorr for 3 h. For secondary drying, the temperature was increased to 15 °C and maintained under 700 mtorr for 10 h. The dried IVIG samples were subsequently stored at 4 °C until used for further analysis. The residual moisture content was determined using a Karl–Fischer titrator (Eco model, Metrohm, Herisau, Switzerland). Approximately 20 mg of dried sample was directly introduced into the titration vessels containing anhydrous Karl–Fischer reagent, with continuous stirring. The water content was determined from the volume of titrant used.

### 2.3. Agitation Stress Test

The agitation stress test was carried out to evaluate the stability of IVIG formulations under mechanical and interfacial stress conditions commonly encountered during handling and transportation. All samples were subjected to end-over-end rotation in a multi-mixer (Seoulin Bioscience, Seoul, Republic of Korea) at 99 rpm (F1 mode; end-over-end) for 5 days. During the agitation period, the multi-mixer was placed inside an incubator maintained at 25 °C. IVIG samples (10 mg/mL), both with and without additives, were exposed to these agitation conditions. All samples were evaluated both before and after agitation stress. Identical analyses were performed post-agitation, including aggregate content, size distribution, and subvisible particles (>2 µm, >10 µm, and >25 µm), using size-exclusion chromatography (SEC), dynamic light scattering (DLS), and flow-imaging microscopy (FI), respectively.

### 2.4. Size-Exclusion Chromatography (SEC)

The samples were filtered by centrifuging through a sterile 0.22 µm cellulose acetate Spin-X filter unit (Costar, Corning Inc., Corning, NY, USA) at 13,000 rpm for 30 s before analysis. Size-exclusion chromatography was performed using an Agilent 1260 system with UV detection at 280 nm. An analytical column (TSKgel G3000SWXL, 30 cm × 7.8 mm, 5 µm, 25 nm pore size; Tosoh Bioscience, Tokyo, Japan) was maintained at 30 °C, and three-times-concentrated phosphate-buffered saline containing approximately 411 mM NaCl, 8.1 mM KCl, 30 mM Na_2_HPO_4_, and 5.4 mM KH_2_PO_4_ and total phosphate ~35 mM (pH adjusted to 7.4) served as the mobile phase, at a flow rate of 0.5 mL/min for 30 min. The injection volume was 20 µL, and all samples were stored in the autosampler at 4 °C before injection. The monomer content was quantified using a calibration curve. At the same time, high-molecular-weight (HMW) species were determined by calculating the ratio of the summed area of pre-main peaks to the combined area of pre-main and monomer peaks, expressed as a percentage. HMW species were defined as all peaks eluting prior to the monomer peak in the size-exclusion chromatogram. Although absolute quantification was not performed using an external calibration curve, all chromatograms were processed using an automated and consistent baseline correction method, and peak areas were integrated within predefined retention time limits, enabling reliable relative comparison across samples.

### 2.5. Flow-Imaging Microscopy (FI)

Samples collected before and after agitation were analyzed using a FlowCam 8100 system with a 10× magnification camera (Yokogawa Fluid Imaging Technologies, Inc., Scarborough, ME, USA) to quantify subvisible particles. Before each measurement, the cleanliness of the fluid path and flow cell was verified by running deionized water, ensuring that background counts were <100 particles (p)/mL. For each measurement, 1 mL of IVIG sample solution was loaded into the sample loader. The initial 0.2 mL was utilized for system priming, and data acquisition was performed on the subsequent 0.2 mL of sample. Particle size determination was based on the area-based diameter (ABD), and data analysis was conducted using Visual Spreadsheet software (version 4.0, Fluid Imaging Technologies).

### 2.6. Dynamic Light Scattering (DLS)

The hydrodynamic size and diffusion parameters of IVIG sample solutions were determined using a Zetasizer Nano ZS90 (Malvern Instruments, Malvern, UK). For particle size analysis, 300 µL of each sample was loaded into UV-transparent disposable cuvettes (ZEN0118, Malvern Instruments, UK). The Z-average diameter, polydispersity index (PDI), and diffusion coefficient were recorded for each measurement. Measurements were conducted at 15 °C, with five consecutive measurements performed for each sample. For the calculation of the diffusion interaction parameter (*k_D_*), 200 µL aliquots of IVIG prepared at concentrations of 2 mg/mL, 4 mg/mL, 6 mg/mL, 8 mg/mL, and 10 mg/mL containing amino acids or their acetyl derivatives were analyzed at a 90° scattering angle. All measurements were carried out in triplicate, and diffusion coefficients were measured at each specified concentration. The *k_D_* value was determined by the slope of the graph plotting diffusion coefficient (*D*) against IVIG concentration (*C*), using the equation *D* = *D*_0_(1 + *k_D_ × C*), where *D*_0_ represents the diffusion coefficient at infinite dilution. For zeta potential analyses, 900 µL of 10 mg/mL IVIG containing 150 mM additives was transferred into a DTS1070 cuvette (Malvern Instruments, UK). Each measurement was repeated five times per sample.

### 2.7. Heat-Induced Protein Aggregation

Thermally induced aggregation was investigated using dynamic light scattering under controlled temperature ramp conditions. Samples (200 µL) were placed in 3 × 3 mm quartz cuvettes (ZEN0118, Malvern Instruments, UK) and equilibrated at 25 °C prior to measurement. For stepwise analysis, measurements were performed at a fixed scattering angle of 90° while the temperature was increased to 70 °C at controlled rates of 5 °C/min to assess concentration-dependent aggregation behavior. For the onset temperature of aggregation (*T_agg_*), samples were subjected to a continuous temperature ramp, from 25 to 70 °C at a rate of 1 °C/min. *T_agg_* was defined as the temperature at which the initial deviation from baseline scattering intensity was observed. Scattering intensity and hydrodynamic diameter were monitored throughout the experiment to evaluate aggregation onset and kinetics as a function of heating rate. Hydrodynamic diameters were calculated using the Zetasizer software v8.02 which applies temperature-dependent solvent viscosity corrections in the Stokes–Einstein equation when water is selected as the dispersant.

### 2.8. Viscosity Measurement

Before analysis, 1 mL aliquots of amino acid buffers were added to the vials to reconstitute the lyophilized samples, which were then allowed to hydrate for at least 30 min. The viscosity of 200 mg/mL IVIG samples formulated with different compositions was measured after equilibrating samples at 25 °C using an m-VROC II viscometer (RheoSense Inc., San Ramon, CA, USA) at a shear rate of 2000 to 3000 s^−1^ equipped with a B05 chip (RheoSense Inc.). The samples were drawn into 100 μL Hamilton syringes and measured nine times. The measurements were carried out automatically at flow rates optimized for the corresponding shear rates.

## 3. Results and Discussion

### 3.1. Effect of Amino Acid Acetylation on Protein–Protein Interaction

Protein–protein and protein–solvent interactions govern the colloidal stability of protein solutions and can be evaluated through the concentration dependence of protein diffusivity [25,26]. The *k_D_* quantifies these interactions: positive *k_D_* values indicate predominant repulsive interactions, whereas negative values indicate attractive interactions, resulting in an increased risk of protein association and aggregation [23,26,27]. The *k_D_* values for IVIG, in the presence and absence of additives, are presented in Table 1. IVIG without additives exhibited the highest positive *k_D_* value, indicating a low propensity for protein aggregation, consistent with previous reports [23]. This observation is in agreement with the relatively high positive zeta potential (18 mV) observed under the same conditions, suggesting strong electrostatic repulsion among protein molecules (Table 1).

In contrast, the addition of 150 mM amino acids bearing an α-amino group (arginine hydrochloride, histidine hydrochloride, and lysine hydrochloride) resulted in a negative shift in *k_D_* values (−5.87 mL/g, −8.17 mL/g, and −9.59 mL/g, respectively), indicating net attractive interactions and reduced colloidal stability. This trend was consistent with their lower zeta potentials (7.04 mV, 6.34 mV, and 6.01 mV, respectively), reflecting decreased electrostatic repulsion under acidic conditions at 25 °C. In comparison, samples containing NA-Arg, NA-His, and NA-Lys at the same concentration exhibited positive *k_D_* values (6.83 mL/g, 16.22 mL/g, and 3.11 mL/g, respectively) along with higher zeta potentials (13.40 mV, 17.57 mV, and 9.46 mV, respectively), suggesting enhanced electrostatic stabilization. Overall, a consistent relationship between *k_D_*, zeta potential, and aggregation behavior was observed, with higher *k_D_* values corresponding to increased zeta potential, supporting the role of electrostatic interactions in governing protein aggregation. The enhanced stability observed with acetylated amino acids may be attributed to increased electrostatic repulsion, as reflected by higher zeta potential values, which can reduce attractive protein–protein interactions.

Comparable findings have been previously reported, demonstrating a conversion of the initially positive *k_D_* of IgG to negative values following the addition of 150 mM arginine hydrochloride [19,23]. Previous studies using single-protein systems, such as bovine serum albumin, have demonstrated that arginine salts can influence translational diffusion through electrostatic screening and modulation of intermolecular interactions [28]. These observations illustrate general protein physicochemical principles; however, the magnitude and manifestation of such effects in IVIG are governed by its polyclonal composition and are therefore evaluated directly in the present study using IVIG-specific measurements. It is plausible that at pH 4, amino acids in the hydrochloride salt form carry net positive charges, markedly increasing the ionic strength of the solution and influencing protein–protein interactions and electrostatic properties [29]. An increase in ionic strength decreases the Debye length, thereby screening electrostatic repulsion between protein molecules and reducing colloidal stability [30]. Specific side-chain interactions, such as guanidinium-mediated cation-π associations of arginine, are well documented in proteins and provide attractive noncovalent contributions. The role of counter-ions (Cl^−^), which may also intensify the attractive forces, provides a mechanistic explanation for the negative shift in *k_D_* observed [31].

**Figure 1:**
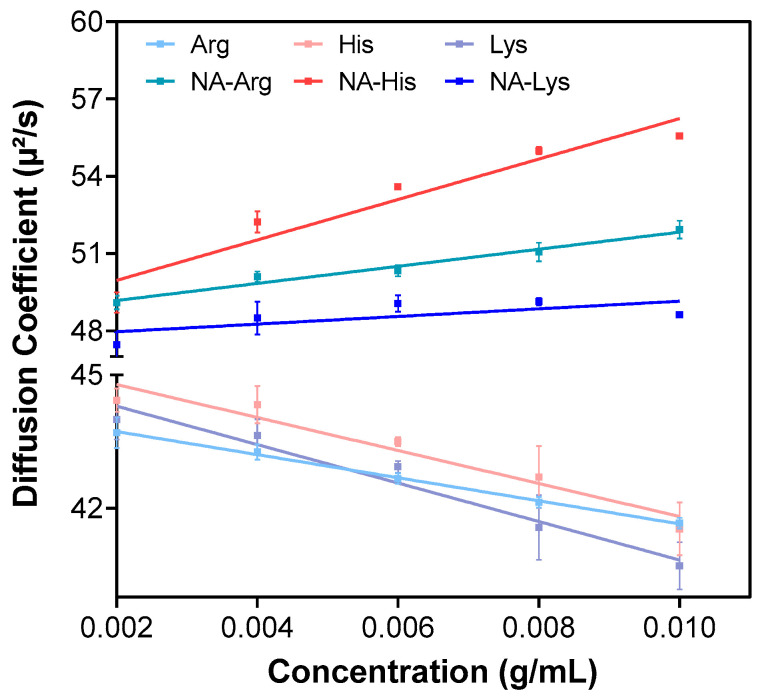
Best-fit analysis of the diffusion coefficient of IVIG samples containing 150 mM amino acids or their acetylated derivatives over a concentration range of 0.002–0.01 g/mL. The diffusion coefficient increased with decreasing concentration in samples containing N-acetylated amino acids, whereas the non-acetylated samples exhibited the opposite trend.

On the other hand, the three derivatives that lack an α-amino group, specifically their N-acetylated forms, generally preserved positive *k_D_* values. Notably, N-acetyl-histidine demonstrated a positive *k_D_* in addition to a strongly positive zeta potential (17.57 ± 0.96 mV), and its diffusion coefficient increased as concentration increased (Figure 1). These results suggest that chemically modifying the α-amino group by N-acetylation neutralizes the positive charge of the backbone amine, thus diminishing weak attractive interactions while strengthening net repulsive protein–protein interactions, leading to greater colloidal stability compared to non-acetylated (or natural) amino acids [19].

### 3.2. Heat-Induced Protein Aggregation

**Figure 2:**
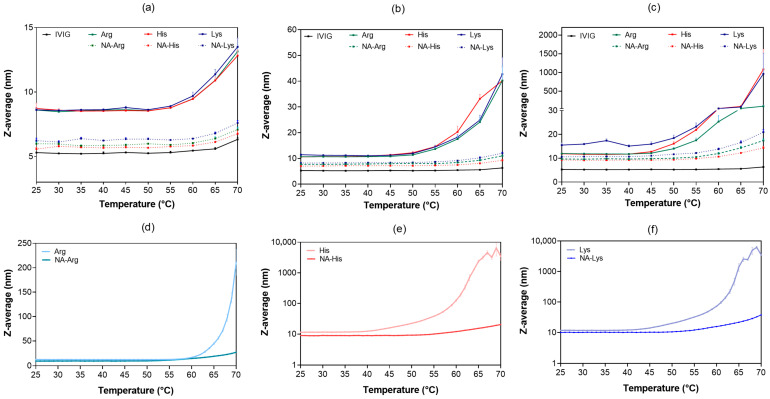
Assessment of Z-average size of 10 mg/mL IVIG containing different concentrations of additives, measured over a temperature range from 25 °C to 70 °C at pH 4 with a heating ramp of 5 °C/min: (**a**) 10 mM, (**b**) 50 mM, and (**c**) 150 mM. Z-average size of 10 mg/mL IVIG measured with a finer temperature ramp (1 °C/min) in the presence of 150 mM (**d**) arginine hydrochloride, (**e**) histidine hydrochloride, and (**f**) lysine hydrochloride, along with comparison to their acetylated derivatives.

DLS evaluated the aggregation characteristics of IVIG as temperature increased to determine the effects of amino acids and their acetyl derivatives. Firstly, the temperature was increased from 25 °C to 70 °C, and particle size analysis was performed at every 5 °C increment (Figure 2a–c). With an increase in concentration from 50 mM to 150 mM, there was a drastic increase in hydrodynamic size in the formulation of IVIG containing histidine and lysine hydrochloride (Figure 2c). To determine the effect of heat and aggregation point precisely, all samples with 150 mM additives were re-evaluated with increasing heat by 1 °C. The hydrodynamic sizes measured by DLS reflect temperature-dependent protein self-association behavior. Despite applying solvent viscosity corrections, higher temperatures may contribute to changes in the apparent z-average due to increased molecular mobility. IVIG at pH 4 showed only an increase in Z-average size, from 5.19 nm at 25 °C to 9.52 nm at 70 °C, indicating limited self-association under acidic conditions.

All acetylated amino acids exhibited greater suppression of aggregation size increase during heating than their unmodified counterparts. The particles generated ranged from 27 nm to 38 nm and lacked any abrupt transition, indicating a robust capacity to inhibit protein self-association. In contrast, arginine hydrochloride yielded particles of approximately 212 nm, whereas lysine and histidine hydrochlorides produced markedly larger aggregates near 6000 nm (Figure 2d–f). A pronounced shift in Z-average size was evident near 45 °C for lysine and histidine, characteristic of aggregation onset, while their acetylated counterparts exhibited delayed transitions closer to 55 °C. These results are consistent with *k_D_* values and reinforce the conclusion that acetylation alleviates disruptive charge interactions, thereby sustaining a smaller particle size distribution post-heating [32]. In acidic environments, the presence of an acetylated group reduced IgG protein–protein interactions by minimizing changes in ionic strength and maintaining surface charge repulsion, resulting in decreased monomer–aggregate and aggregate–aggregate associations as previously reported in IVIG with acetylated arginine [23]. Amino acids that retained the α-amino group displayed a tendency for molecular clustering, with their charge states under acidic conditions promoting a larger particle size distribution, indicating the formation of insoluble aggregates (i.e., >100 nm).

The gradual change in slope or apparent increase in the temperature response reflects early, reversible self-association or changes in collective diffusion behavior rather than an increase in intrinsic thermal stability [33,34]. As temperature increases, reduced long-range electrostatic repulsion can increase protein–protein collision frequency, promoting intermolecular association and accelerating aggregation kinetics under destabilizing conditions [35]. Similar temperature-dependent increases in apparent hydrodynamic size have been observed in the IVIG with amino acids and are correlated with protein aggregation propensity [19,23]. Furthermore, heat-induced aggregation of high-concentration antibodies may indicate irreversible protein aggregation [6].

### 3.3. Agitation-Induced Protein Aggregation

**Figure 3:**
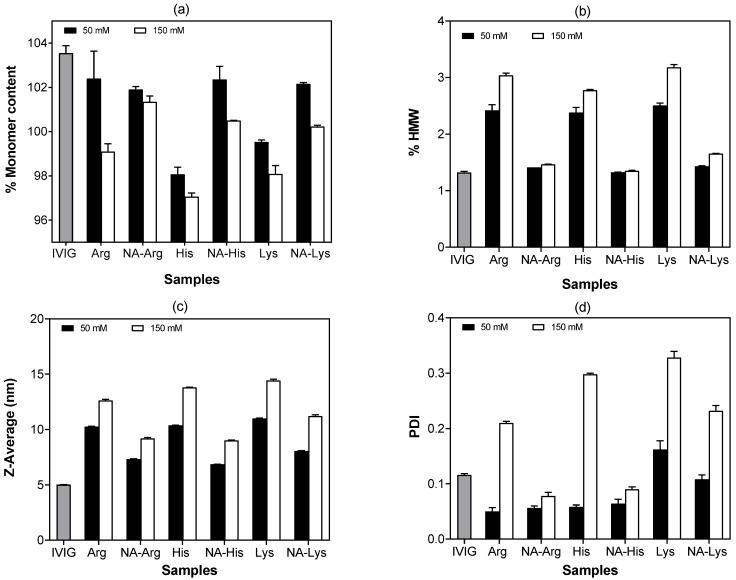
Bar graphs representing SEC results: (**a**) monomer content (%) and (**b**) HMW (%). Each sample contained 10 mg/mL IVIG with or without amino acid additives following 5 days of agitation at 25 °C. DLS measurements for 10 mg/mL IVIG showing (**c**) Z-average size (nm) and (**d**) PDI in 25 mM sodium acetate buffer containing 50 mM and 150 mM additives at pH 4 after agitation. Data are presented as mean ± SD (*n* = 5), highlighting reduced size distribution and heterogeneity following N-acetylation. Gray bars indicate IVIG before the addition of additives.

**Figure 4:**
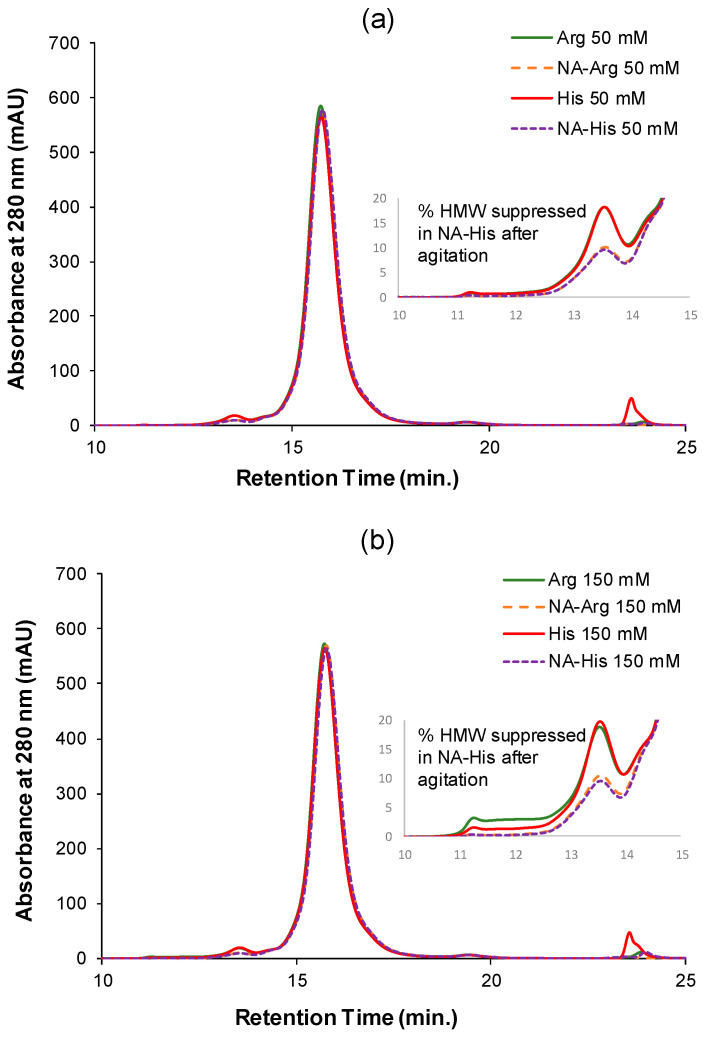
Overlaid SEC chromatograms of 10 mg/mL IVIG containing (**a**) 50 mM and (**b**) 150 mM Arg, NA-Arg, His, and NA-His in 25 mM sodium acetate buffer (pH 4) after agitation.

Agitation stress testing was conducted to assess the stabilizing properties of acetylated amino acids in IVIG formulations. IVIG samples (10 mg/mL) supplemented with 50 mM and 150 mM additives underwent end-over-end rotation at 25 °C for 5 days. Interestingly, an apparent increase in monomeric content was observed after agitation (Figure 3a). This behavior has been reported previously for IVIG and is attributed to the dissociation of weak, reversible protein–protein associations during SEC analysis rather than the absence of aggregation [19,23]. In addition, monomer content was quantified by SEC peak area integration using a calibration curve (i.e., values may exceed 100%). As a result, enhanced electrostatic repulsion limits the formation of soluble aggregates (i.e., <100 nm), while transient self-associated species generated during agitation can readily dissociate upon dilution and chromatographic separation, resulting in high apparent monomer recovery. Accordingly, SEC results are interpreted as reflecting preservation of chemical integrity, whereas reversible aggregation and particle formation are more sensitively captured by complementary techniques such as DLS and FI. NA-His and NA-Arg consistently maintained the highest monomer content and demonstrated the lowest aggregate formation (Figure 3b). Overlaid SEC chromatograms suggest that soluble aggregates eluted before monomers, with NA-His showing the lowest HMW fraction (Figure 4).

DLS analysis was also performed along with SEC to provide complementary characterization of species after agitation stress. The particle size distributions and polydispersity profiles (Figure 3c,d) demonstrated that all samples containing acetylated amino acids had notably lower Z-average size than their parent amino acids at matched concentrations. In comparison, 150 mM of arginine hydrochloride, lysine hydrochloride, and histidine hydrochloride led to a broader size distribution and greater size heterogeneity. It is well established that the addition of salts modifies the electrical double layer surrounding proteins through electrostatic screening by counterions and co-ions, thereby influencing the apparent hydrodynamic size measured by DLS [28,36]. At acidic pH, amino acids in their hydrochloride form are predominantly protonated and contribute substantially to ionic strength, with histidine hydrochloride exerting a particularly strong effect [37,38,39]. An increase in ionic strength reduces electrostatic repulsion between protein molecules, which can promote intermolecular interactions and lead to an increase in apparent hydrodynamic diameter [40]. Furthermore, DLS intensity plots revealed the presence of several large-sized peaks in samples with parent amino acids, while N-acetyl derivatives mostly showed a single, narrow peak around 10 nm (later discussed). Taken together, these results indicate that ionic strength is a dominant contributor to the observed z-average trends, and acetylation may mitigate interaction-driven clustering under comparable conditions, reducing apparent hydrodynamic heterogeneity [41,42].

**Figure 5:**
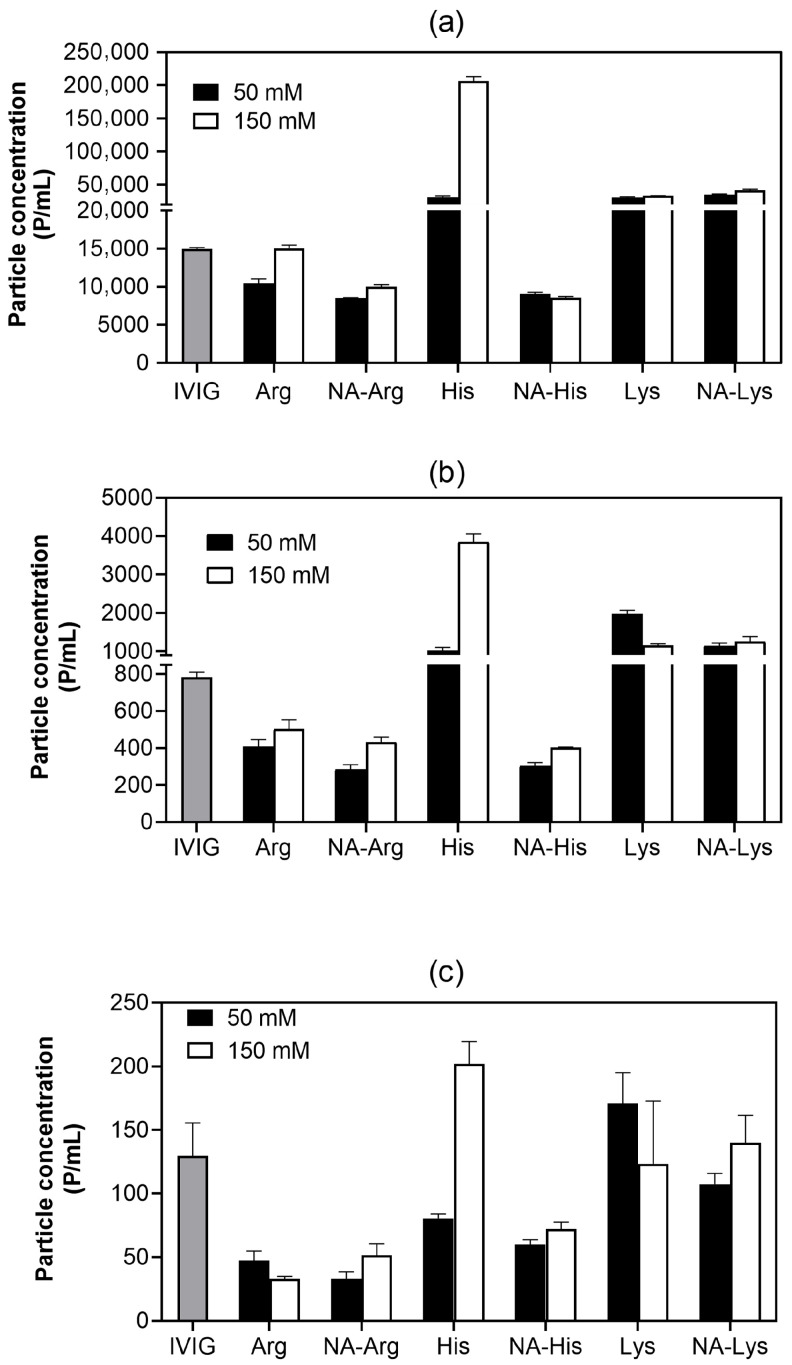
Bar graphs illustrating subvisible particle counts in the following size ranges: (**a**) 2–10 µm, (**b**) 10–25 µm, and (**c**) >25 µm in samples formulated in 25 mM acetate buffer. Samples were subjected to 5 days of agitation at 25 °C. Results are presented as mean ± SD (*n* = 3). Gray bars indicate IVIG before the addition of additives.

Subvisible particles (SVP, i.e., >2 μm) were further quantified using FI employing the area-based diameter (ABD) [43]. Figure 5 includes the control (IVIG without amino acids), enabling direct comparison between baseline particle levels and additive-dependent effects. The no-additive IVIG control yielded approximately 15,000 p/mL (2 to 10 µm) after agitation. The particle level is consistent with previous studies reporting that residual glycine present in commercial IVIG formulations can partially suppress weak, reversible aggregation under acidic conditions [19,44]. In contrast, amino acid salts, except arginine hydrochloride at 50 mM, generated SVP levels that exceeded baseline levels, indicating exacerbation of particle formation under agitation stress. The corresponding N-acetylated amino acids consistently suppressed SVP formation relative to their parent salts and, in certain cases, relative to the no-additive control. At 150 mM, NA-His and NA-Arg yielded around 8600 p/mL and 10,000 p/mL, respectively, compared to 206,600 p/mL for histidine hydrochloride. This divergence was more pronounced in the larger particle fraction (≥25 µm), where acetylated amino acids markedly reduced agitation-induced larger particle formation relative to both the parent salts and the control.

**Figure 6:**
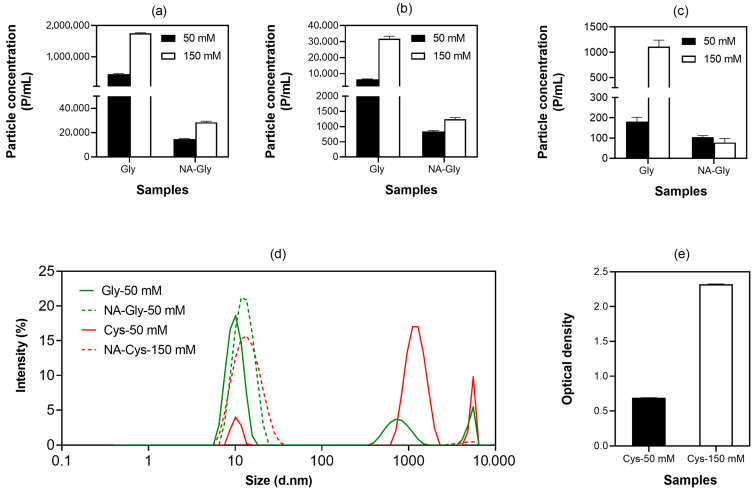
Comparison of subvisible particle counts in the following size ranges: (**a**) 2–10 µm, (**b**) 10–25 µm, and (**c**) >25 µm for samples formulated in 25 mM acetate buffer containing glycine hydrochloride and its acetylated derivative. Samples were subjected to 5 days of agitation at 25 °C. Results are presented as mean ± SD (*n* = 3). (**d**) Intensity-based particle size distributions of 10 mg/mL IVIG with 50 mM additives. (**e**) Optical density of samples containing cysteine hydrochloride at 50 and 150 mM after agitation. FI images further demonstrated that larger particles were more prevalent in formulations containing parent amino acids, whereas their abundance was reduced in acetylated analogues. Flow imaging (FI) was used as a descriptive technique to characterize particle size and morphology, including shape factor, aspect ratio, and irregularity, which varied depending on the excipients used. Formulations containing parent amino acids produced particles with lower circularity and higher elongation, indicating irregular and heterogeneous aggregates with increased surface area and structural perturbation (Figure 7) [43,47]. In contrast, formulations with N-acetyl arginine and N-acetyl histidine produced more spherical particles, with circularity values closer to 1, indicating more compact and uniform structures. Such irregular or fibrous morphologies may enhance interactions with the immune system and have been associated with increased immunogenicity-related responses, including inflammasome activation and cytokine release [48,49]. Conversely, more spherical and less elongated particles are generally associated with improved structural integrity, enhanced stability, and reduced immunogenicity potential [47]. Consistent with previous reports, complement activation is largely independent of particle morphology within the subvisible size range [46]. The concurrent increases in Z-average size, polydispersity index, and oligomer content quantified by DLS and SEC, together with FI observations, are therefore interpreted as indicative of early-stage aggregation processes rather than definitive mechanistic pathways leading to visible particle formation [50]. In parallel, a lower zeta potential (closer to zero) with amino acid associated with reduced electrostatic repulsion can facilitate protein–protein interactions and promote aggregation [35]. Multiple alternative mechanisms—including kinetic effects during particle formation, solvent–protein and solvent–excipient interactions, and variations in nucleation and growth dynamics—may contribute to the observed particle morphologies and were not independently resolved in this study [51,52].

On the other hand, additional amino acids—cysteine and glycine in their hydrochloride forms, along with their acetyl derivatives—were subjected to agitation under the same conditions. Visual examination showed the presence of precipitation in IVIG containing cysteine hydrochloride after agitation, as indicated by optical density readings at 350 nm of 0.69 and 2.32 at 50 mM and 150 mM, respectively (Figure 6e). Acetyl-cysteine remained clear with a lower SVP level. Also, glycine hydrochloride increased the SVP level with increasing concentration in all size ranges by more than ten times compared with acetyl-glycine (Figure 6a–c). The presence of multiple peaks in the DLS particle size distribution indicates the presence of larger particles with cysteine hydrochloride and glycine hydrochloride (Figure 6d). As a result, the presence of acetylated amino acids facilitates protein stabilization under stress conditions. Agitation is known to enhance turbulent mixing and collision frequency, increasing protein–protein interaction and partial unfolding, thereby promoting the size of protein aggregates [45,46]. Acetylated amino acids more effectively maintained colloidal dispersion and suppressed particle formation.

### 3.4. Viscosity of IVIG with Acetylated Amino Acids

**Figure 8:**
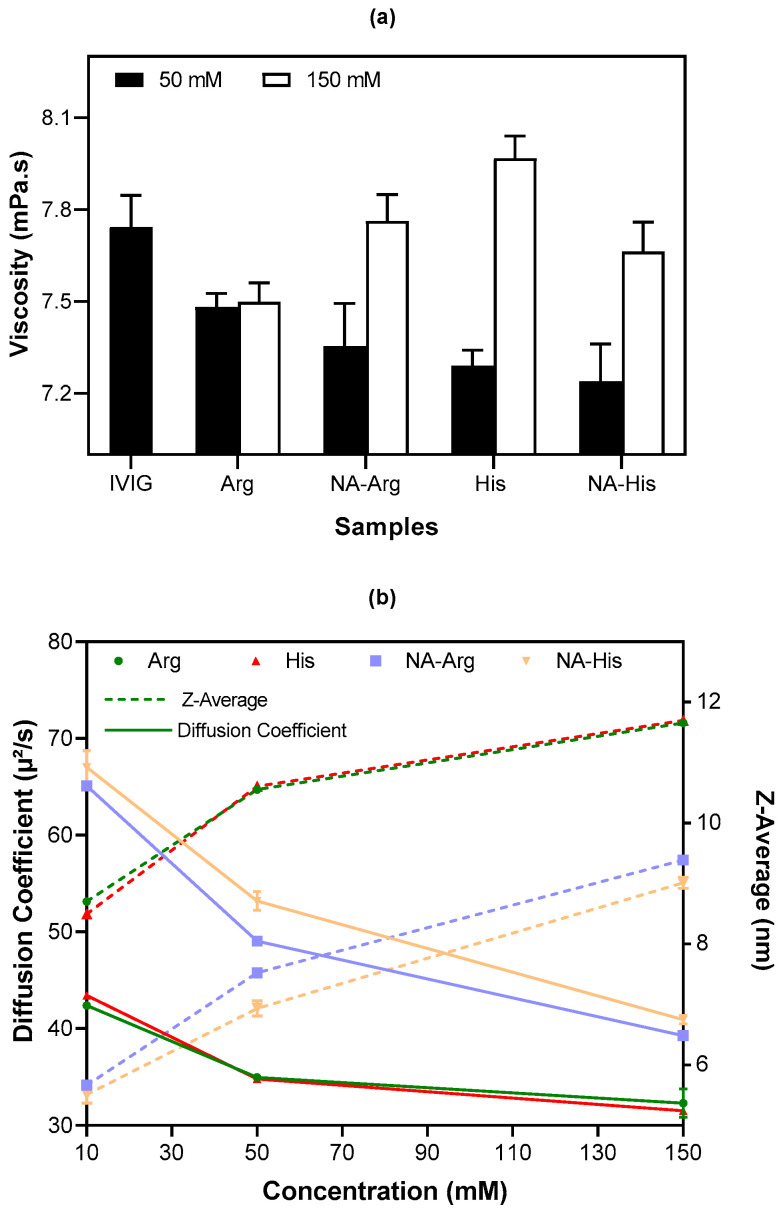
(**a**) Average apparent viscosity (mPa·s) of 200 mg/mL IVIG in the presence of 50 and 150 mM Arg, His, and their acetylated derivatives after reconstitution for 30 min. Data are presented as mean ± SD (*n* = 9). (**b**) Diffusion coefficient (µm^2^/s) and Z-average size (nm) of 10 mg/mL IVIG samples containing 10 mM, 50 mM, and 150 mM additives.

High protein concentration enhances intermolecular interactions and contributes to aggregation propensity in solution. Arginine hydrochloride, histidine hydrochloride, and their corresponding acetylated derivatives reduced aggregation under the tested conditions. Viscosity was also examined to characterize formulation behavior. Following lyophilization, the residual water content in IVIG was measured at 6.83 ± 0.97%. The retained monomer content was determined by SEC, yielding 94.27% relative to pre-lyophilization samples. The mean viscosity of reconstituted 200 mg/mL IVIG was 7.7 mPa·s (Figure 8a). All samples with 50 mM additives exhibited reduced viscosity. Although viscosity changes were modest, pronounced excipient-dependent differences were observed in agitation-induced subvisible particle formation. Furthermore, acetylated amino acids exhibited marginally lower viscosity than their parent forms, particularly at low concentrations. The viscosity remained almost unchanged across the tested shear rate range, showing only a slight increase indicative of Newtonian-like flow behavior, with negligible evidence of shear-thinning or thickening effects [53]. All tested formulations exhibited viscosities below commonly accepted injectability limits (<25 cp) at shear rates of 2000–3000 s^−1^, indicating suitability for injectable administration. However, high-concentration protein formulations (>100 mg/mL IVIG) are known to present viscosity-related challenges, including increased injection force (>10–20 N), reduced injectability, and processing limitations [54]. Viscosity was therefore evaluated as a formulation parameter associated with protein–protein interactions and aggregation behavior. Although the observed differences were modest, they reflect underlying changes in intermolecular interactions that can influence formulation stability and manufacturability. Future work should focus on systematic evaluation of viscosity across a broader range of formulation conditions.

These findings are consistent with the established relationship between the diffusion interaction parameter and viscosity, whereby positive *k_D_* values are associated with lower viscosity, and more negative *k_D_* values correlate with higher viscosity [55]. The hydrodynamic diameter (Z-average) and translational diffusion coefficient of 10 mg/mL IVIG samples with increasing concentration of amino acids were measured using dynamic light scattering (Figure 8b). The Zetasizer software v8.02 derives the diffusion coefficient from the autocorrelation function and converts it to the z-average using the Stokes–Einstein relationship, based on the input temperature and viscosity [56]. With increasing concentrations of all amino acids from 10 mM to 150 mM, an increase in the Z-average was observed, indicating the formation of larger aggregates. This was accompanied by a decrease in the diffusion coefficient (D), reflecting reduced molecular mobility in solution. According to the Stokes–Einstein relation, the observed decrease in diffusion coefficient reflects increases in hydrodynamic size and/or solution viscosity. In addition, samples containing acetylated amino acids exhibited relatively higher diffusion coefficients (>40 µm^2^/s) and smaller Z-average values (~9 nm) compared with their corresponding parent amino acids, which showed diffusion coefficients < 35 µm^2^/s and Z-average values ~11.5 nm at higher concentrations (Figure 8b). These results suggest that aggregation is associated with reduced molecular mobility and may contribute to increased viscosity through enhanced intermolecular interactions and hydrodynamic volume. Consistent with previous studies, arginine hydrochloride also decreased the viscosity of highly concentrated protein solutions [17,57]. Its effect was maintained at both 50 mM and 150 mM. In comparison, the other prepared samples exhibited concentration-dependent increases in size, SVP levels, and viscosity. As a result, 50 mM acetylated amino acids would be beneficial for stabilizing protein formulations. Given that histidine is already widely used as a buffering agent, its acetylated form may warrant further investigation as a formulation component. At the mechanistic level, acetylation may influence electrostatic interactions, but this effect appears to be amino acid-specific and concentration-dependent, requiring further study.

### 3.5. Future Perspectives

**Figure 9:**
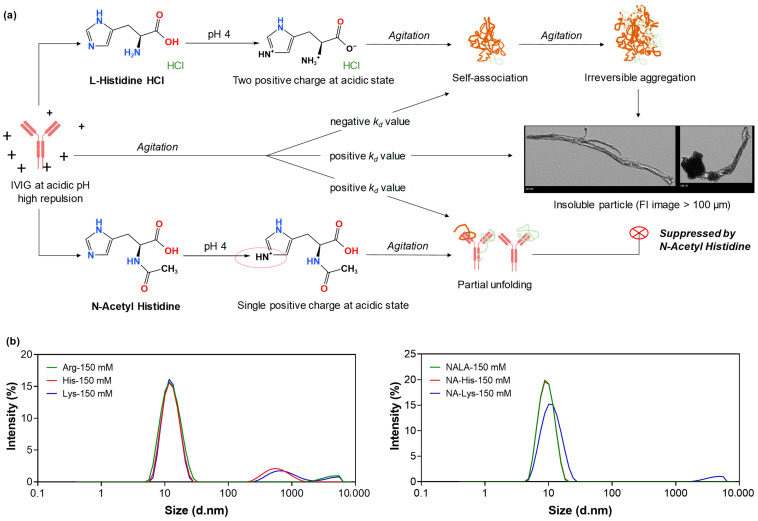
(**a**) Schematic overview of the study design and comparative effects of acetylated amino acids in high-concentration protein formulations. (**b**) Intensity-based particle size distributions of 10 mg/mL IVIG with 150 mM additives.

Figure 9a offers a schematic overview of the present study in accordance with the current working hypothesis. Upon exposure to thermal and agitation stress, IVIG formulations containing acetylated forms of arginine hydrochloride, histidine hydrochloride, and lysine hydrochloride suppressed sub- or visible particles and exhibited a narrow particle size distribution along with a decreased count of large particles (Figure 9b). In contrast, lysine and its acetylated form demonstrated lower aggregation suppression and displayed stronger attractive forces than those observed in arginine- and histidine-based additives. DLS data further indicated that 50 mM acetylated histidine and arginine facilitated greater repulsive forces, more rapid diffusion, and a reduced propensity for aggregation, findings that were suggested by FI particle analysis. Importantly, these findings demonstrate that chemical modification of the α-amino group can systematically alter protein–protein interaction landscapes in a stress-dependent manner, rather than identifying N-acetylation itself as a universal stabilizing solution. In this context, N-acetylation serves as a representative proof-of-concept modification, illustrating that targeted chemical modification of the α-amino group provides a rational handle for tuning amino acid-mediated stabilization mechanisms in protein formulations [58,59].

As a caveat, protein stability in therapeutic formulations is governed by both intrinsic and extrinsic factors, including pH, ionic strength, temperature, mechanical and interfacial stresses, as well as the protein’s inherent physicochemical characteristics [60,61,62,63,64]. Protein aggregation poses a persistent obstacle, as it may compromise product stability, trigger immune responses, heighten hypersensitivity, and lead to dose inaccuracies or reduced biological function [65,66,67,68,69,70]. While acetylated histidine (NA-His) demonstrated favorable buffering capacity and colloidal stabilization under acidic conditions in the present study, these results should be interpreted as evidence supporting the broader design principle of α-amino group modification rather than as endorsement of a single optimal excipient.

## 4. Conclusions

The present study demonstrates that N-acetylated amino acids function as interaction-modulating excipients that alter aggregation behavior and subvisible particle formation in a stress-dependent manner in IVIG formulations. 50 mM acetylated amino acids consistently influenced interaction-driven instability, particularly where agitation-induced particle formation was suppressed. Viscosity measurements of reconstituted high-concentration IVIG indicated formulation-dependent differences, highlighting that the impact of amino acids on colloidal behavior is strongly governed by ionic strength and surface charge. In this context, not only N-acetylated arginine but also N-acetylated histidine exhibited favorable interaction-modulating behavior under acidic conditions. Overall, this work establishes that chemical modification of the α-amino group provides a rational strategy for tuning amino acid-mediated protein interactions, offering a conceptual framework for the future development of next-generation protein formulation stabilizers.

## Figures and Tables

**Figure 7:**
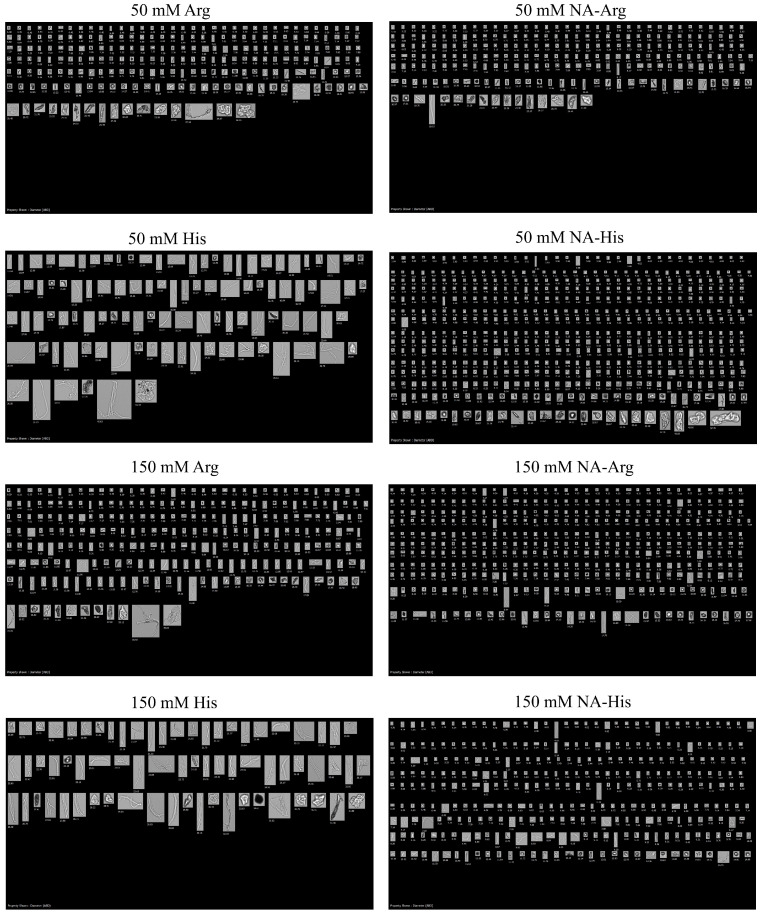
Representative FI images of particles formed after agitation in 10 mg/mL IVIG with 50- and 150-mM additives in sodium acetate buffer (pH 4).

**Table 1:** *k_D_*, *T_agg_*, and Zeta potential values of IVIG in the presence of 150 mM amino acids and their derivatives evaluated by DLS.

Protein	+ Additives	*k_D_* (mL/g)	*T_agg_* (°C)	Zeta Potential (mV)
IVIG only	-	111.27	67	18.40 ± 1.60
	Arg	−5.87	61	7.04 ± 0.53
	NA-Arg	6.83	61	13.40 ± 0.66
	His	−8.17	43	6.34 ± 0.45
	NA-His	16.22	60	17.57 ± 0.96
	Lys	−9.59	46	6.01 ± 1.09
	NA-Lys	3.11	63	9.46 ± 1.30

## Data Availability

The original contributions presented in this study are included in the article. Further inquiries can be directed to the corresponding authors.
